# The maximum entropy model optimized with the kuenm package predicts the potential habitat distribution of *Aconitum pendulum* in China under climate change

**DOI:** 10.3389/fpls.2026.1844124

**Published:** 2026-06-19

**Authors:** Bing He, Jiamin Ruan, Digao Wan, Mazhong Ban, Deji Duo, Jiangbin Zhong, Xiao Guo, Qien Li

**Affiliations:** 1 College of Pharmacy, Qinghai University, Xining, China; 2 College of Clinical Medicine, Qinghai University, Xining, China; 3 College of Ecological Environmental Engineering, Qinghai University, Xining, China; 4 Tibetan Medicine Research Center of Qinghai University, Tibetan Medical College, Qinghai University, Xining, China

**Keywords:** *Aconitum pendulum*, climate change, environmental variables, habitat prediction, maximum entropy model

## Abstract

**Introduction:**

*Aconitum pendulum* is a rare medicinal plant endemic to the Qinghai-Tibet Plateau and a key indicator species for alpine ecosystems. It possesses both medicinal and ecological value. However, its wild populations are on the verge of decline due to overharvesting and low reproductive capacity, and the species is highly sensitive to climate change.

**Methods:**

In this research, the kuenm package was employed to optimize the maximum entropy model, so as to predict the distribution of *A. pendulum* in China under both current and future climate scenarios. From a total of 103 environmental variables, 11 key factors influencing the distribution of *A. pendulum* were selected through correlation analysis and variable contribution rate evaluation. Based on 188 valid distribution sites of *A. pendulum*, an analysis was conducted on its potential habitat distribution in China under current and future climatic conditions.

**Results:**

The results demonstrate that under the current climate, the total area of suitable habitats for *A. pendulum* is 2.08×10^6^ km^2^, accounting for 21.56% of China’s land area, and these habitats are mainly distributed in the southwest, northwest, and central regions of the country. Altitude, september solar radiation, and temperature seasonality were identified as the main factors affecting the distribution of *A. pendulum*. Under future climate change scenarios, the overall area of suitable habitats for *A. pendulum* exhibits a decreasing trend. Under the low-emission scenario, the area of unsuitable areas shows slight fluctuations, while the low-, medium-, and high-suitable distribution areas within the suitable zones remain balanced. Under the high-emission scenario, the edge areas of unsuitable areas shrink rapidly, whereas the high-suitable areas expand slightly. The centroid migration of the suitable habitats did not deviate from the core region located on the eastern edge of the Qinghai-Tibet Plateau, indicating that *A. pendulum* still has adaptive potential under climate change.

**Discussion:**

This study highlights the significant impacts of altitude, September solar radiation, and temperature seasonality on the distribution of *A. pendulum*, and provides a scientific basis for the protection, planting planning, and sustainable development of *A. pendulum*.

## 1 Introduction

Global climate change has brought about rising temperatures, changed precipitation regimes, and an increase in extreme weather events. These changes have exerted a profound influence on plant physiological metabolism and geographical distribution patterns, resulting in migration, contractions, or expansions of species’ suitable habitats and thus endangering the stability of biodiversity and ecosystems (Habibullah et al., 2022). *Aconitum pendulum*, a perennial herbaceous species, as a rare medicinal plant and ecological indicator species endemic to the Qinghai-Tibet Plateau region of China, it possesses considerable medicinal and ecological significance. In terms of medicinal value, its tuberous roots have long been utilized in traditional Chinese medicine, as they are rich in alkaloids with anti-inflammatory and analgesic effects (Wang et al., 2022; Li et al., 2022), and serve as a crucial raw material for the treatment of rheumatoid arthritis (Zhang et al., 2025). Ecologically, this species mainly dwells in specific habitats including alpine meadows, shrublands, and gravelly slopes, and shows high sensitivity to environmental factors such as climate and soil. As a key indicator species that reflects changes in alpine ecosystems, its robust inverted conical taproot plays an effective role in soil and water conservation, thereby sustaining the stability of alpine ecosystems (Wang, 2023). However, the populations of *A. pendulum* are currently on the verge of decline due to overharvesting, low natural reproductive capacity, long growth cycles, and immature cultivation techniques, resulting in a severe imbalance between supply and demand. Furthermore, as the species is highly sensitive to climate and soil conditions, climate change will further alter its suitable habitat, exacerbating the risk of its extinction (Sun et al., 2024a).

Forecasting the potential habitat distribution of plants is a key tool for mitigating the impacts of climate change on species (Fan et al., 2025). It allows for the accurate identification of habitats conducive to species survival, which provides a scientific foundation for the conservation of species resources, the development of *in situ* and *ex situ* conservation strategies, and the sustainable management of biodiversity. This method is of great significance for alleviating the ecological risks associated with climate change. In research on predicting the suitability of plant ecological distribution, species distribution models have been extensively utilized in plant ecology, conservation biology, and related fields. This is attributed to their capacity to quantify the response relationships between species and environmental factors, thereby forecasting the distribution areas of species (Milanesi et al., 2019; Gonzalez et al., 2011; Engler et al., 2013). Among these models, the maximum entropy (MaxEnt) model has become the effective tool for predicting the habitat distribution of plants. Its widespread application is due to its unique advantages, including low demand for distribution data, high prediction accuracy, strong adaptability, and effective management of small sample sizes (Banavar et al., 2010; Pueyo et al., 2007; Yang et al., 2025). To date, the MaxEnt model has been successfully applied to studies on predicting the suitability distribution of various rare and endangered plants, such as *Aconitum leucostomum* (Xu et al., 2024), *Rhododendron anthopogonoides* (Yang et al., 2024), and *Lagotis macrosiphon* (Ma et al., 2025). This application has provided reliable technical support for the conservation and rational utilization of species resources.

Although the MaxEnt model exhibits remarkable advantages in predicting plant suitability distribution, its default parameter configurations are often plagued by problems such as excessive subjectivity and irrational feature combinations. These issues frequently result in overfitting or underfitting, which impairs the accuracy and reliability of the prediction outcomes. In order to address these problems, the kuenm package, a specialized R toolkit developed for MaxEnt model optimization can effectively improve the model structure through systematic parameter screening, feature selection, cross-validation, and model evaluation. By removing redundant environmental variables and unreasonable parameter settings, this package significantly enhances the accuracy and stability of predictions (Cobos et al., 2019). Up to now, the kuenm package has been applied in research on MaxEnt model optimization, providing a more reliable technical method for accurately forecasting the potential habitat distribution of species (Cedano Giraldo and Mumcu Kucuker, 2024; Zheng et al., 2023; Liu et al., 2025; Li et al., 2024a).

In the context of climate change, changes in environmental factors will further affect the distribution of suitable habitats for *A. pendulum*, which may cause the migration and shrinkage of its suitable living areas and further worsen its endangered situation. Therefore, accurately forecasting the potential habitat distribution of *A. pendulum* in China under climate change, and clarifying the spatial pattern and future variation trends of its suitable habitats, is of great significance for the *in situ* conservation of its wild resources, the formulation of *ex situ* conservation schemes, the selection of artificial cultivation sites, and the sustainable utilization of its resources. Based on these considerations, by optimizing the MaxEnt model with the kuenm package and integrating data on current and future climate change scenarios, the potential habitat distribution of *A. pendulum* in China under climate change was predicted. The spatial distribution characteristics and change patterns of its suitable habitats were analyzed, providing a scientific basis for the scientific conservation and rational utilization of *A. pendulum*.

## 2 Materials and methods

### 2.1 Collection and processing of distribution data for *A. pendulum*

**Figure 1:**
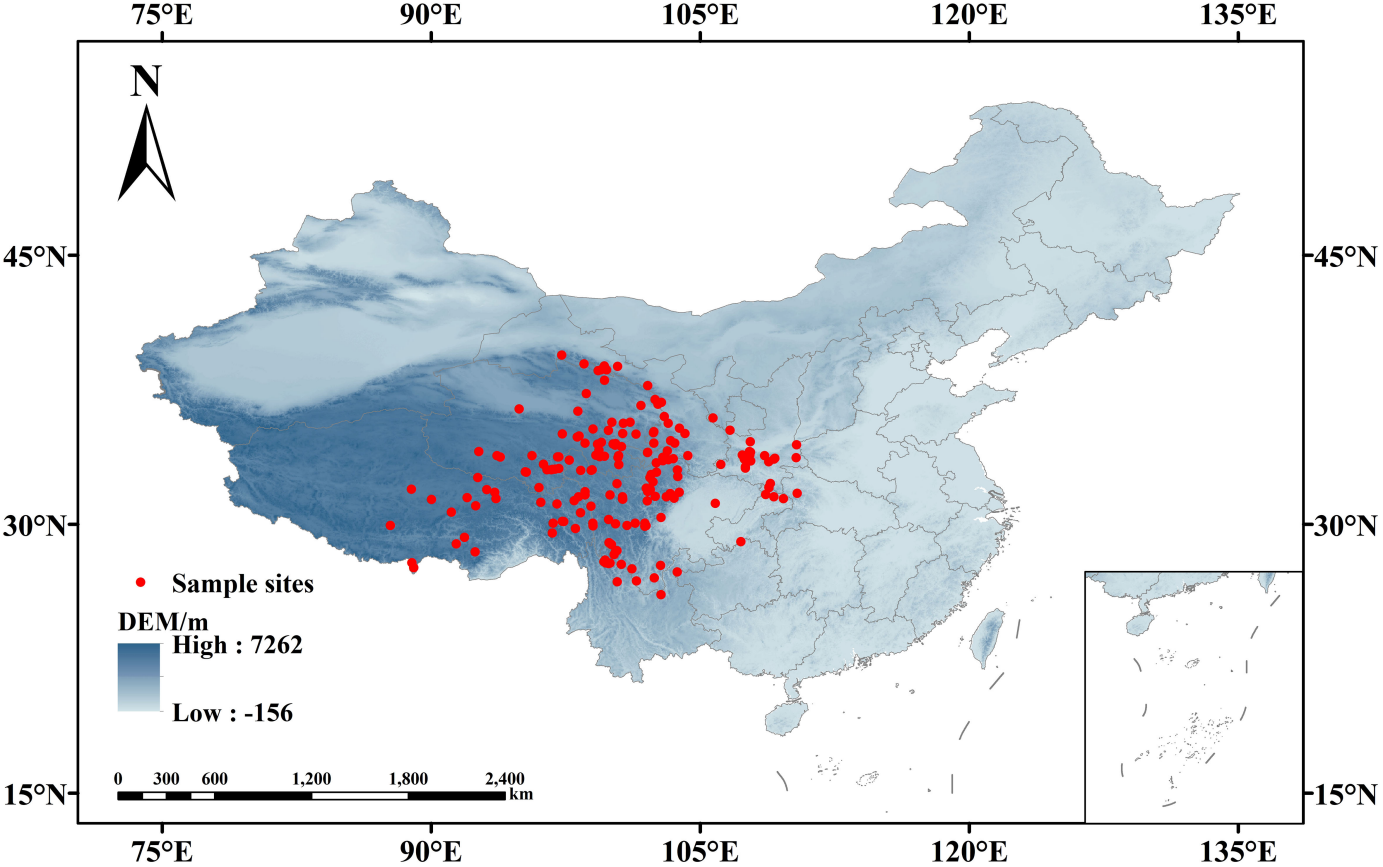
Spatial distribution of *A. pendulum* in China.

Occurrence records of 204 A*. pendulum* individuals documented after 1970 were gathered. These distribution data were obtained from two authoritative platforms: the National Specimen Information Infrastructure (http://www.nsii.org.cn/) and the China Virtual Herbarium (https://www.cvh.ac.cn/). To mitigate spatial autocorrelation, the SDM tools software was utilized to remove redundant data points, with the aim of ensuring that only one distribution point was retained within each 1 km×1 km grid cell (Crase et al., 2014; Brown et al., 2017). This data pruning process ultimately resulted in 188 valid distribution points, and their specific geographical locations are illustrated in **Figure 1**.

### 2.2 Sources and processing of environmental data

The environmental datasets utilized in this research, encompassing both current and future climate data, were retrieved from the WorldClim database (https://worldclim.org/). This dataset included 19 bioclimatic variables (bio1 - bio19), in addition to maximum temperature, minimum temperature, average temperature, monthly solar radiation and precipitation. Elevation, slope, and aspect were derived from the Digital Elevation Model, while soil texture data were obtained from the Harmonized World Soil Data. Spatial demographic datasets were sourced from WorldPop. The future climate projection data were founded on the Coupled Model Intercomparison Project Phase 6, specifically adopting the Beijing Climate Center Climate System Model (BCC-CSM2-MR) (Dong et al., 2025). Emissions scenarios based on shared socioeconomic pathways (SSPs) were examined, covering four time periods (2021-2040, 2041-2060, 2061-2080, and 2081-2100) under two extreme scenarios (SSP126 and SSP585). The spatial resolution of all 103 environmental variables was standardized to 2.5 minutes (**Table 1**).

**Table 1:** Detailed information of 103 environment variables.

Variable code	Environmental factor
tmin 1-12	January to December minimum temperature
tmax 1-12	January to December maximum temperature
tavg 1-12	January to December average temperature
prec 1-12	January to December precipitation
srad 1-12	January to December solar radiation
bio1	Annual Mean Temperature
bio2	Mean Diurnal Range
bio3	Isothermality
bio4	Temperature Seasonality
bio5	Max Temperature of Warmest Month
bio6	Min Temperature of Coldest Month
bio7	Temperature Annual Range
bio8	Mean Temperature of Wettest Quarter
bio9	Mean Temperature of Driest Quarter
bio10	Mean Temperature of Warmest Quarter
bio11	Mean Temperature of Coldest Quarter
bio12	Annual Precipitation
bio13	Precipitation of Wettest Month
bio14	Precipitation of Driest Month
bio15	Precipitation Seasonality
bio16	Precipitation of Wettest Quarter
bio17	Precipitation of Driest Quarter
bio18	Precipitation of Warmest Quarter
bio19	Precipitation of Coldest Quarter
alt	Altitude
slope	Slope
aspect	Aspect
zbyl	Vegetation Classification
coarse	Coarse fragments
sand	Sand
slit	Slit
clay	Clay
bulk	Bulk Density
ref_bulk	Reference Bulk Density
org_cbn	Organic Carbon Content
pH	pH in water
n	Total nitrogen content
cn	Carbon/Nitrogen ratio (C/N)
cec_soil	Cation Exchange Capacity Soil
cec_clay	Cation Exchange Capacity clay
teb	Total Exchangeable Bases
bsat	Base Saturation
alum_sat	Aluminium saturation
esp	Exchangeable Sodium Percentage
eq	Calcium Carbonate
gypsum	Gypsum content
elec_con	Electric Conductivity
pop2020	Population Distribution

Given the potential for strong collinearity among environmental variables, the following protocol was implemented to filter 103 variables and identify the key factors for MaxEnt model construction (Chen et al., 2025a): Initially, these variables were imported into ENMTools software to compute the Pearson correlation coefficients (|r|) for every pair of variables. Subsequently, all 103 environmental variables were integrated with the 188 distribution records and subjected to preliminary MaxEnt runs to quantify the contribution rate of each variable to the model. Based on a dual-criteria screening strategy, variables were retained if they met two conditions: a correlation coefficient threshold of |r| < 0.8 was applied, and the variable exhibited a contribution rate exceeding 0.4 in the preliminary MaxEnt model. Following this rigorous selection, a total of 11 key environmental variables were finalized: alt, bsat, bulk, cec_clay, ref_bulk, slit, bio_3, bio_4, prec_09, srad_05, and srad_09.

### 2.3 Development, optimization, and evaluation of the MaxEnt model

The processed distribution data of *A. pendulum*, together with the filtered environmental factors, were introduced into the MaxEnt v3.4.4 software (Phillips et al., 2006). Specific parameter configurations for the model were set as follows: 25% of the data were allocated as the test set, while 75% were used as the training set. The Bootstrap approach was adopted, with the default maximum number of background points set to 10,000. Random seed was activated to guarantee the reproducibility of the model results. Additionally, the output format was specified as logistic values, and the number of repetitions was set to 10.

The MaxEnt model comprises five distinct feature types: linear (L), quadratic (Q), hinge (H), product (P), and threshold (T). Given that the predictive performance of the MaxEnt model is affected by the regularization multiplier (RM) and feature combinations (FC), the kuenm package was employed to optimize 31 distinct feature combinations for the model. The RM was set to range from 0.6 to 4 with an interval of 0.2, yielding 18 distinct values in total. Through the kuenm package, a total of 558 parameter combinations were evaluated. Model performance was assessed using receiver operating characteristic curves and corrected akaike information criterion corrected (AICc) values to screen out the optimal model, whose parameters were subsequently applied to the MaxEnt model.

### 2.4 Data processing for the MaxEnt model

To further explore the changes in the suitable habitat area of *A. pendulum* under current and future climate scenarios, ArcGIS Desktop v10.7.1 (Environmental Systems Research Institute, Inc, 2019) was employed to visualize the species’ suitable habitats. The Maximum Test Sensitivity and Specificity (MTSPS) threshold was adopted for classifying suitable areas (Kaivanto, 2008). Following the import of the average results from the MaxEnt model into ArcGIS Desktop, the Reclassify tool was utilized to divide the suitable areas into four categories: unsuitable areas (0 - MTSPS, MTSPS = 0.2037), low-suitable areas (MTSPS - 0.5), medium-suitable areas (0.5 - 0.7), and high-suitable areas (0.7 - 1). Subsequently, the distribution area of each suitability class was calculated through counting the number of grids in each category.

The current and future suitable habitats of *A. pendulum* were divided into two types: unsuitable areas (0 - MTSPS) and suitable habitats (MTSPS - 1). The distribution of current suitable habitats was overlaid with that of future suitable habitats via the “Intersect” function in ArcGIS Desktop. On the basis of this overlay analysis, the future distribution of *A. pendulum*’s suitable habitats was categorized into three scenarios: range expansion, no change, and range contraction. The geometric centroid of the suitable habitat was defined as the central point of its distribution. The migration of *A. pendulum*’s geometric centroid under the SSP126 and SSP585 scenarios was calculated using the zonal geometry statistics tool in ArcGIS Desktop (Lenoir et al., 2008; Cai et al., 2025).

## 3 Results

### 3.1 Model optimization and evaluation

**Figure 2:**
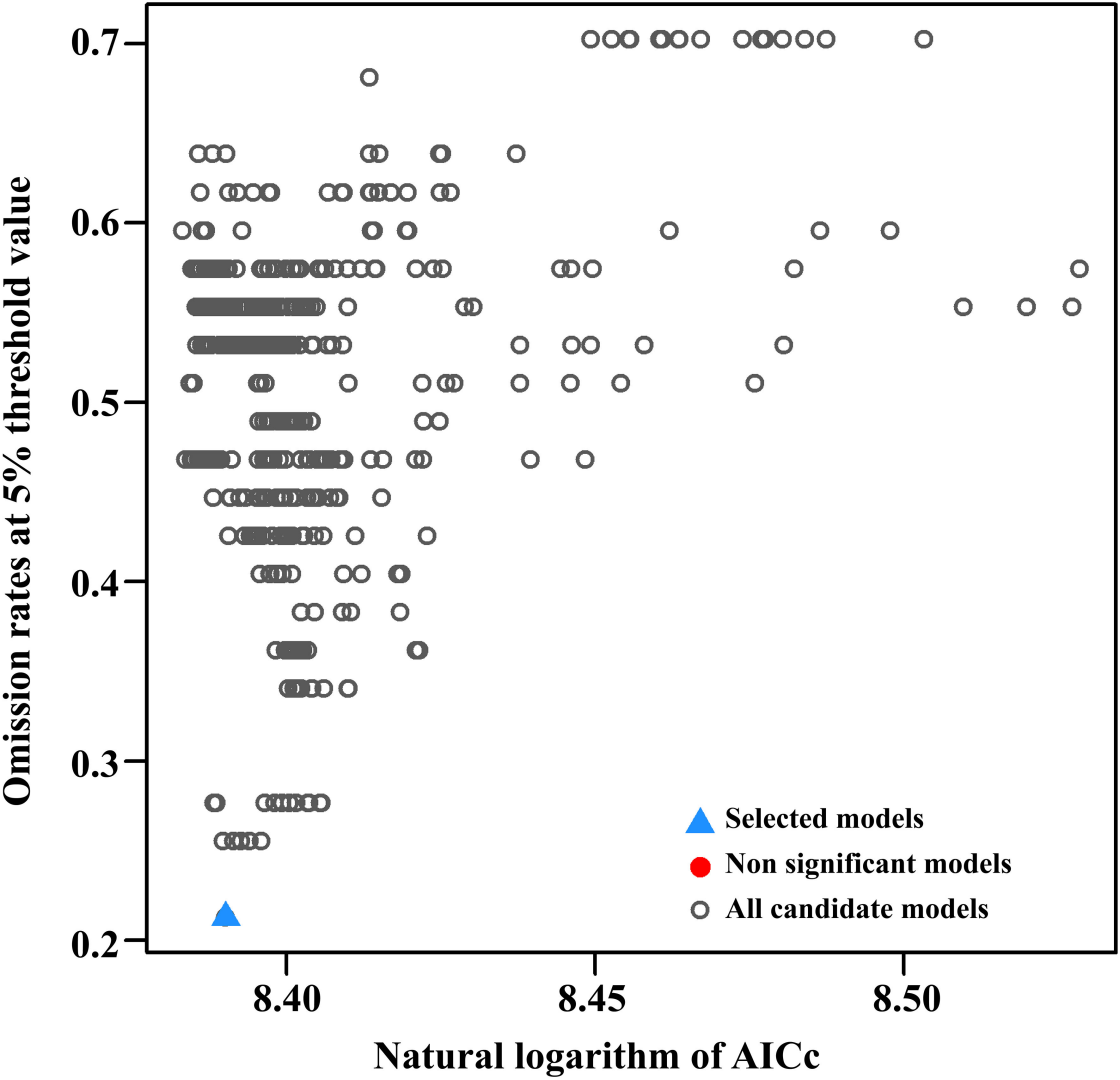
Parameter optimization results of MaxEnt model.

**Figure 3:**
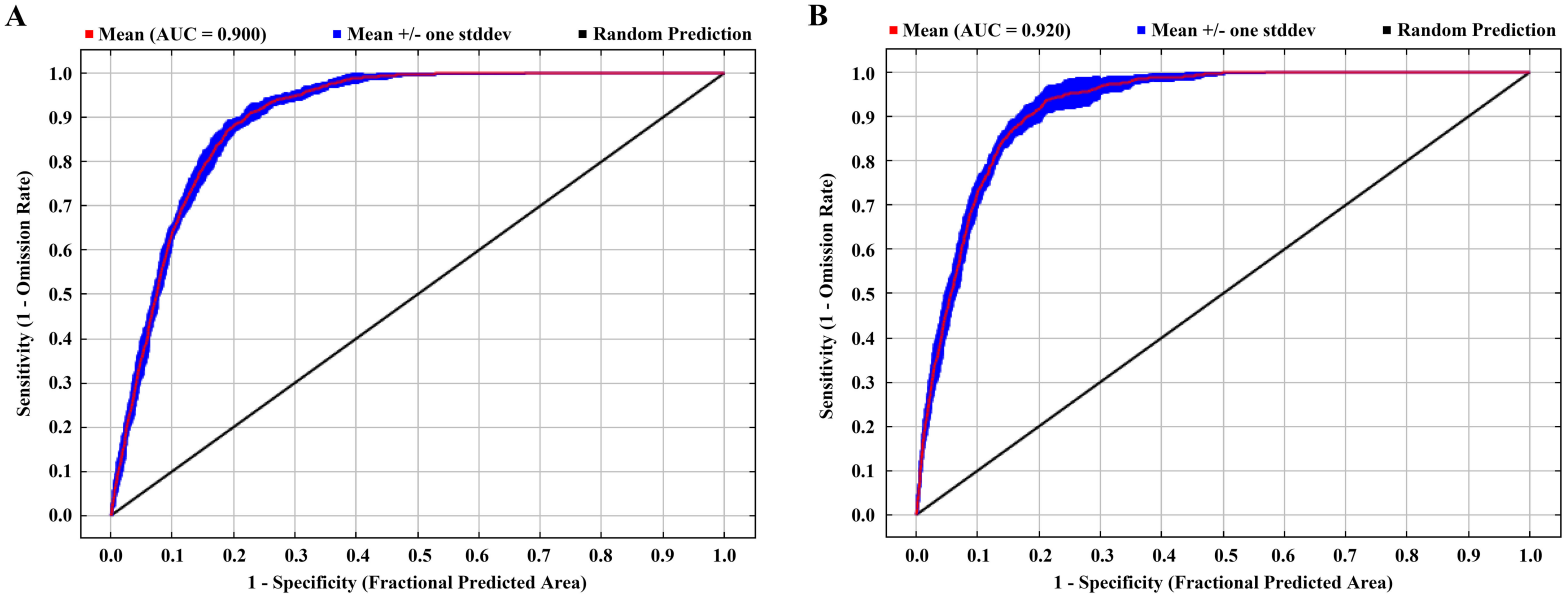
Receiver operating characteristic (ROC) curves for the kuenm package before and after optimization [**(A)** ROC curve before optimization; **(B)** Optimized ROC curve].

When adopting the default parameter settings (FC = LQPH and RM = 1), the AICc value was 4618.5968, with a ΔAICc value of 245.5899. After optimizing the model via the kuenm package and selecting the parameters FC = LQ and RM = 1.2, the AICc value was reduced to 4403.5457, while the ΔAICc value dropped to 0, which signifies the optimal model (**Figures 2**, **3**). Consequently, in this study, the parameter settings FC = LQ and RM = 1.2 were chosen as the final parameters for the *A. pendulum* distribution model.

### 3.2 Impact of key environmental variables on the distribution of *A. pendulum*

**Figure 4:**
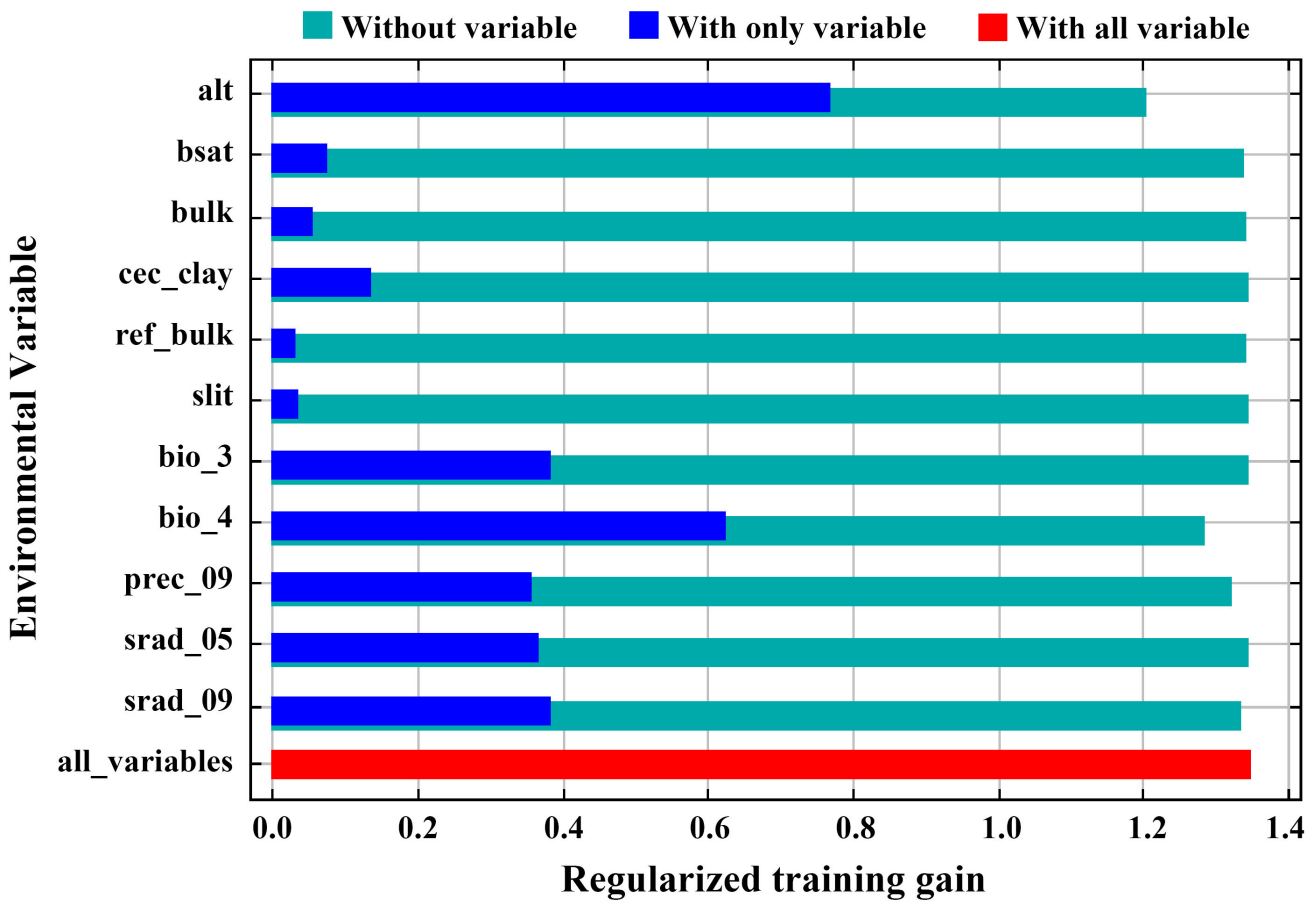
Results of jackknife test for key variables in MaxEnt model.

A total of 11 key environmental variables were employed in the analysis to assess their contributions to the distribution of *A. pendulum*. According to their contribution rates, alt (35.5%), srad_09 (29.2%), and bio_4 (17.7%) were recognized as the main factors for model establishment, while the total contribution of other environmental variables with relatively lower contribution rates accounted for 17.6% (**Table 2**). In addition, the Jackknife method was adopted to further verify the importance of the environmental variables alt, srad_09, and bio_4 (**Figure 4**). Thus, alt, srad_09, and bio_4 were determined to be the primary environmental variables affecting the suitable distribution of *A. pendulum*.

**Table 2:** Contribution rates of key environmental variables.

Variable	Percent contribution (%)	Permutation importance (%)
alt	35.5	49.4
srad_09	29.2	5.9
bio_4	17.7	22.2
cec_clay	6.8	1.2
srad_05	4.7	2.3
bsat	1.8	14.5
bio_3	1.5	1.6
prec_09	0.8	0.3
slit	0.7	1.2
bulk	0.7	1
ref_bulk	0.5	0.4

**Figure 5:**
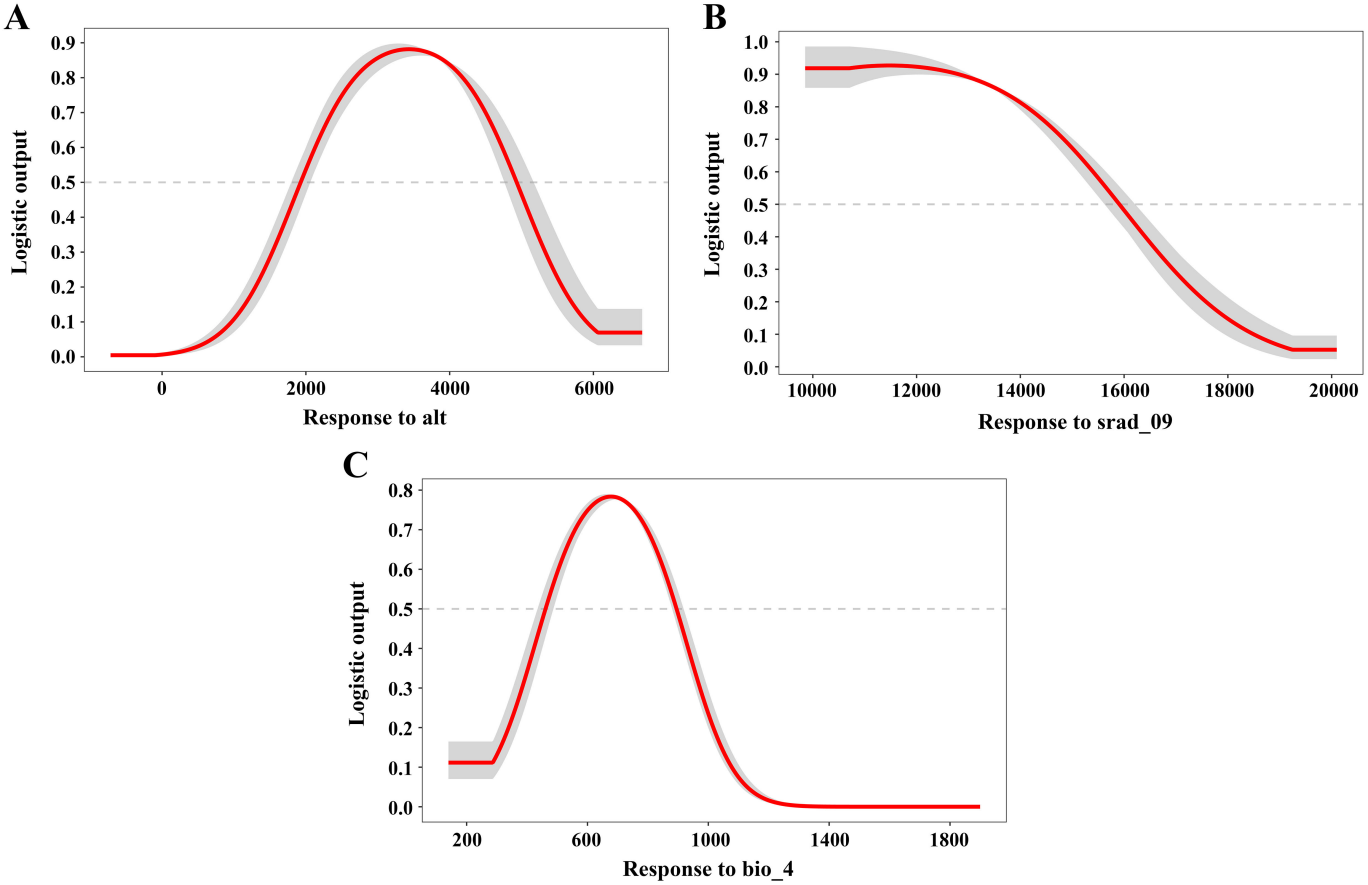
Response curves of key environmental variables that affect the suitable distribution of *A. pendulum* [**(A)** response curves of alt; **(B)** response curves of srad_09; **(C)** response curves of bio_4].

To generate visualizations of the response curves for key environmental variables, logistic output values greater than 0.5 were taken to signify that the corresponding environmental factor is beneficial to plant growth. The results indicated that the most favorable conditions for the survival of *A. pendulum* are an altitude ranging from 1,930.9952 m to 4,918.3328 m, September solar radiation between 9,853.2 kJ·m^-2^·day^-1^ to 15,898.104 kJ·m^-2^·day^-1^, and temperature seasonality (standard deviation × 100) in the range of 463.7632 to 893.0871 (**Figure 5**).

### 3.3 Suitable distribution of *A. pendulum* under current climatic conditions

**Figure 6:**
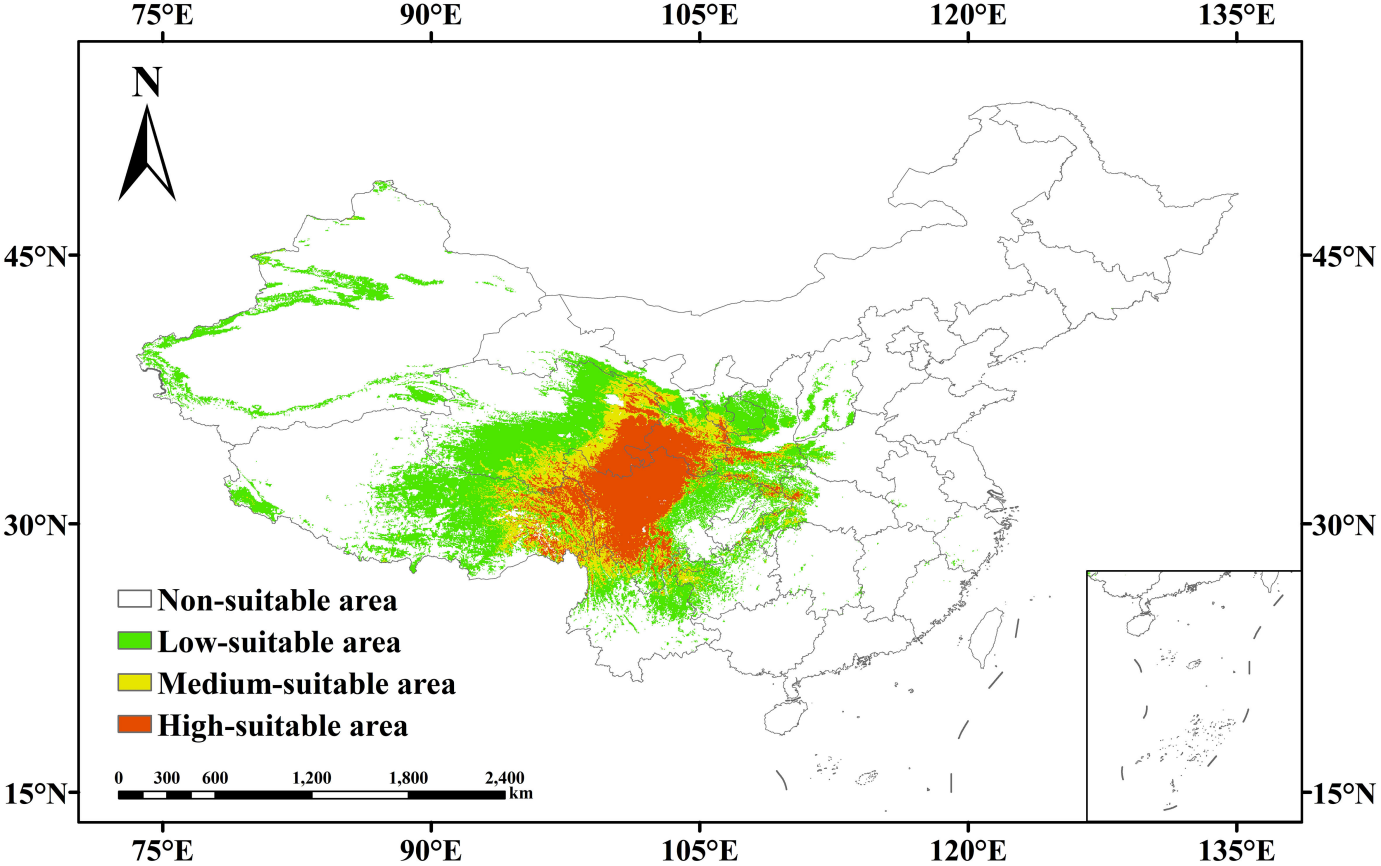
The suitable distribution area of *A. pendulum* under current climate conditions.

Under present climatic conditions, the total suitable habitat area of *A. pendulum* is roughly 2.08×10^6^ km^2^, representing approximately 21.56% of China’s total land area, with a main distribution across southwestern, northwestern, and central parts of the country (**Figure 6**). High-suitable areas were concentrated in the northeastern portion of southwestern China (Sichuan and adjacent areas), the southeastern part of northwestern China (southern Gansu and southern Shaanxi), and western sections of central China (western Hubei). These core habitats were mainly clustered along the transitional zone between the eastern Qinghai-Tibet Plateau and the Sichuan Basin, with an area of 4.8×10^5^ km^2^, accounting for 5% of China’s land area. Medium-suitable areas were primarily distributed in southwestern China (Sichuan, northern Yunnan, and western Guizhou), eastern parts of northwestern China (southern Gansu and eastern Qinghai), and western central China (Chongqing and western Hubei), covering 3.8×10^5^ km^2^, equivalent to 3.96% of the national land area. Low-suitable areas occupied the largest spatial extent, occurring mainly in southwestern China (eastern Xizang, Yunnan, and western Guizhou) and northwestern China (western Xinjiang, most of Qinghai, and western Gansu), with a distribution extending to the western margin of Inner Mongolia. This zone spanned 1.22×10^6^ km^2^, making up 12.71% of China’s total land area.

### 3.4 Optimal distribution of *A. pendulum* under future climate conditions

**Figure 7:**
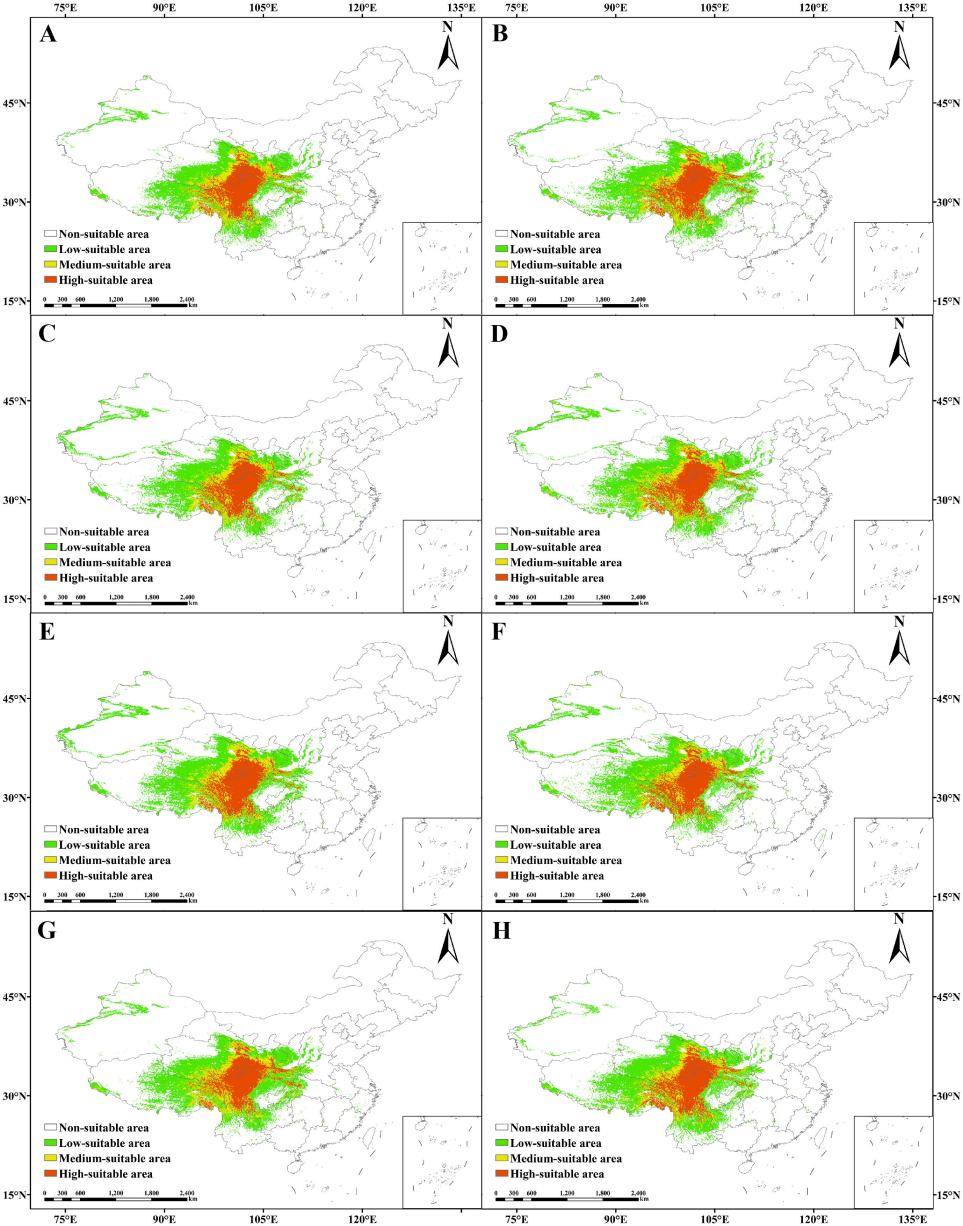
The distribution of suitable habitats for *A. pendulum* under different future climate scenarios SSP126 and SSP585 [**(A–D)** the suitable habitat distribution of *A. pendulum* for SSP126 in 2030s, 2050s, 2070s, and 2090s, respectively; **(E–H)** the suitable habitat distribution of *A. pendulum* for SSP585 in 2030s, 2050s, 2070s, and 2090s, respectively].

On the basis of the optimized MaxEnt model, the MTSPS threshold approach was adopted to simulate habitat suitability under two emission scenarios for four future time intervals. Through these simulations, the spatial distribution of future suitable habitats and their hierarchical suitability for *A. pendulum* were identified (**Table 3**; **Figure 7**).

**Table 3:** The suitable habitat areas of *A. pendulum* in future climate under different suitability levels.

Decade scenarios	Predicted area (× 10^4^ km^2^)			
	Low habitat suitability	Medium habitat suitability	High habitat suitability	Total suitable area
Current	122.34	37.57	47.91	207.82
2030s-SSP126	109.19	36.16	44.97	190.32
2050s-SSP126	117.59	35.84	48.19	201.62
2070s-SSP126	114.36	33.13	45.64	193.13
2090s-SSP126	112.71	39.13	48.61	200.45
2030s-SSP585	121.13	35.99	48.74	205.86
2050s-SSP585	123.91	35.09	50.04	209.04
2070s-SSP585	115.23	40.32	46.82	202.37
2090s-SSP585	114.41	36.24	51.67	202.32

Under the SSP126 scenario, the overall suitable habitat area shows a fluctuating trend of initial increase, subsequent decrease, and final slight recovery. Nevertheless, the total suitable area of *A. pendulum* is predicted to shrink relative to the present climate state. The most substantial reduction appears in the 2030s, with the total suitable area declining by 1.75×10^5^ km^2^. Among them, the low-suitable area is projected to decrease by 1.315×10^5^ km^2^, the medium-suitable areas by 1.41×10^4^ km^2^, and the high-suitable areas by 2.94×10^4^ km^2^. By the 2090s, the total suitable area will rebound slightly, with an overall reduction of 7.37×10^4^ km^2^ compared with current conditions. Specifically, the low-suitable areas will decrease by 9.63×10^4^ km^2^, whereas the medium- and high-suitable areas will expand marginally, increasing by 1.56×10^4^ km^2^ and 7×10^3^ km^2^ respectively.

Under the SSP585 scenario, the overall suitable habitat area for *A. pendulum* also shows a general decreasing trend. Only in the 2050s does the total suitable area exceed the current level by 1.22×10^4^ km^2^. In all other future periods, the suitable area remains smaller than at present. By the 2090s, the total suitable habitat is projected to reach its minimum value of 2.0232×10^6^ km^2^, representing a reduction of 5.5×10^4^ km^2^ compared with current climate conditions. The low-suitable areas shrinks most significantly, decreasing by 7.93×10^4^ km^2^, while the medium-suitable areas declines by 1.33×10^4^ km^2^. In contrast, the high-suitable areas expands by 3.76×10^4^ km^2^.

### 3.5 Spatial patterns of potential future distribution of *A. pendulum*

**Figure 8:**
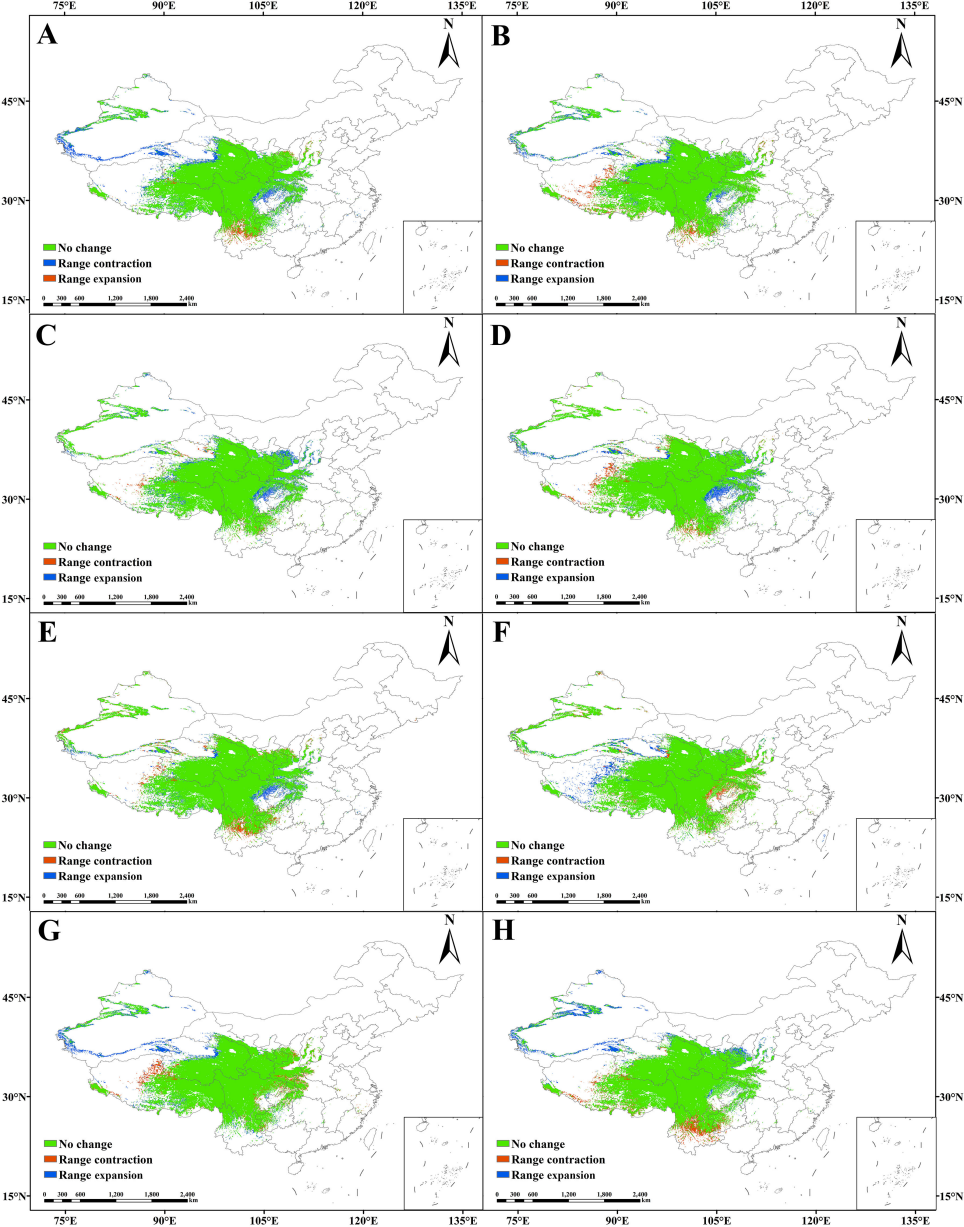
Spatial pattern changes of suitable habitats for *A. pendulum* under different future climate scenarios [**(A–D)** the spatial pattern changes of suitable habitats for *A. pendulum* in SSP585 at 2030s, 2050s, 2070s, and 2090s, respectively; **(E–H)** the spatial pattern changes of suitable habitats for *A. pendulum* in SSP585 at 2030s, 2050s, 2070s, and 2090s, respectively].

**Figure 9:**
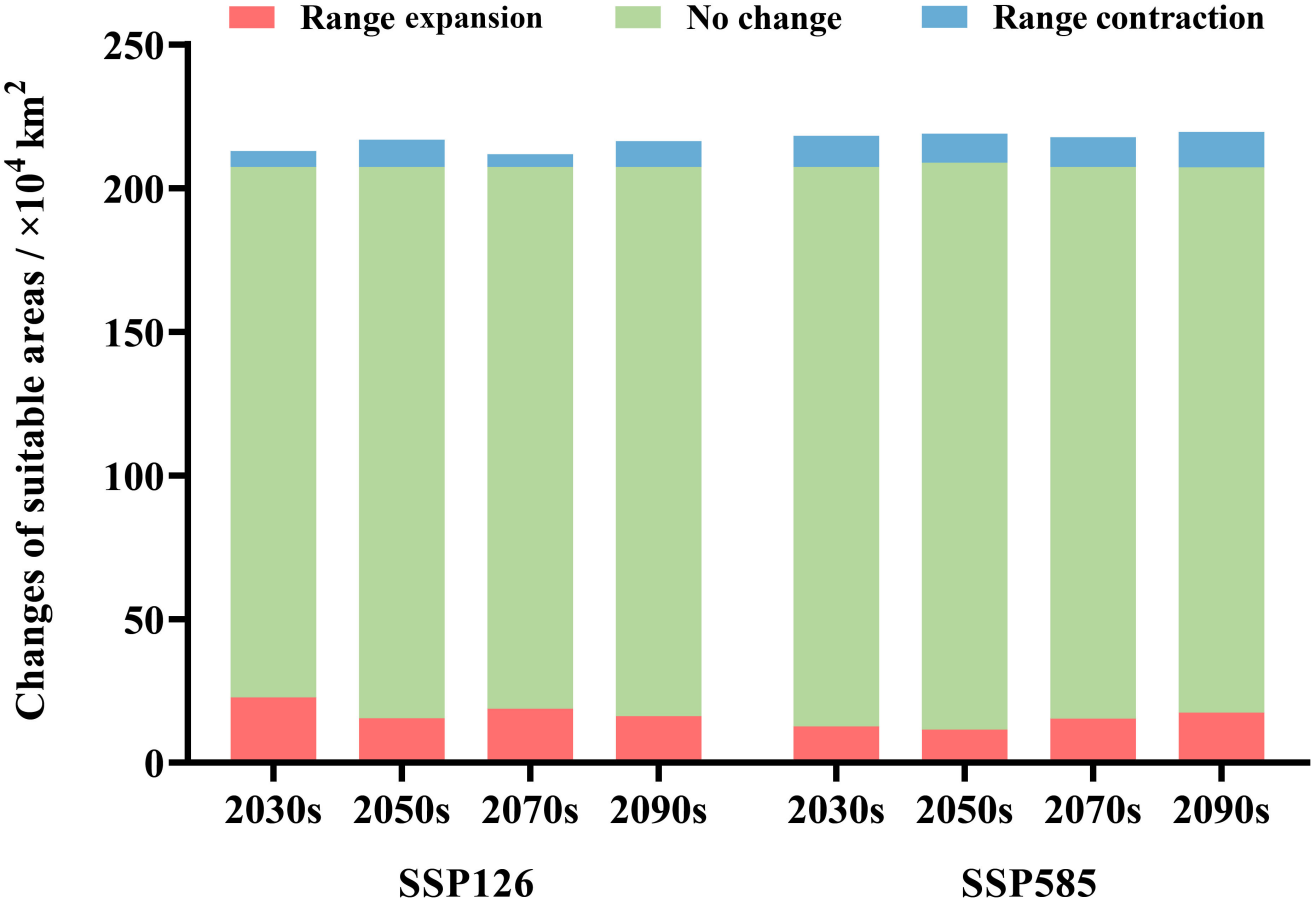
Stacked plot of spatial pattern changes in suitable habitats for *A. pendulum* under different future climate scenarios.

This study compared the potential suitable habitat distribution of *A. pendulum* under future climate scenarios with its current distribution pattern (**Figures 8**, **9**). Findings under the SSP126 scenario reveal that China’s ecosystems present a characteristic pattern of stable core regions with fluctuating peripheral zones. Taking the North China Plain, the Huang-Huai River Basin, and the middle and lower reaches of the Yangtze River as the core areas, these regions consistently make up the largest proportion of the total ecosystem area, with their spatial extent maintaining stability within a range of 1.85×10^6^ km^2^ to 1.92×10^6^ km^2^, covering the major agricultural and ecological core zones of China. Regions experiencing habitat expansion, including northwestern China (Xinjiang and Neimenggu) and northern Northeast China (Heilongjiang and northeastern Neimenggu), displayed a fluctuating trend of “initial decrease, subsequent increase, and final reduction”. In contrast, areas undergoing habitat contraction, such as South China (Guangdong and Guangxi), Southwest China (Yunnan), and the peripheral parts of North China (Hebei and southern Shandong), showed periodic fluctuations in area, ranging from 5.59×10^4^ km^2^ to 9.48×10^4^ km^2^.

Under the SSP585 scenario, ecosystem stability decreases substantially, and spatial responses show more prominent regional heterogeneity, presenting a distribution pattern characterized by “northern expansion, southern contraction, and weakened stability”. The core stable region is identical to that under the SSP126 scenario, but its area displays a trend of “rising first and then declining”, shrinking from 1.95×10^6^ km^2^ to 1.90×10^6^ km^2^. Habitat expansion is mainly concentrated in northern Northeast China (Heilongjiang), with the northwest (Xinjiang) as a secondary expansion region, and the expanded area keeps increasing from 1.26×10^5^ km^2^ to 1.74×10^5^ km^2^. The contraction zones covering South China (Guangdong and Guangxi), Southwest China (Yunnan) and southern North China (Henan and Shandong) expand from 1.08×10^5^ km^2^ to 1.22×10^5^ km^2^. The overall habitat contraction pressure is considerably higher than that under the SSP126 scenario, suggesting a significant intensification of habitat degradation risks under high-emission climate conditions.

### 3.6 Centroid migration of suitable habitats for *A. pendulum* in the future

**Figure 10:**
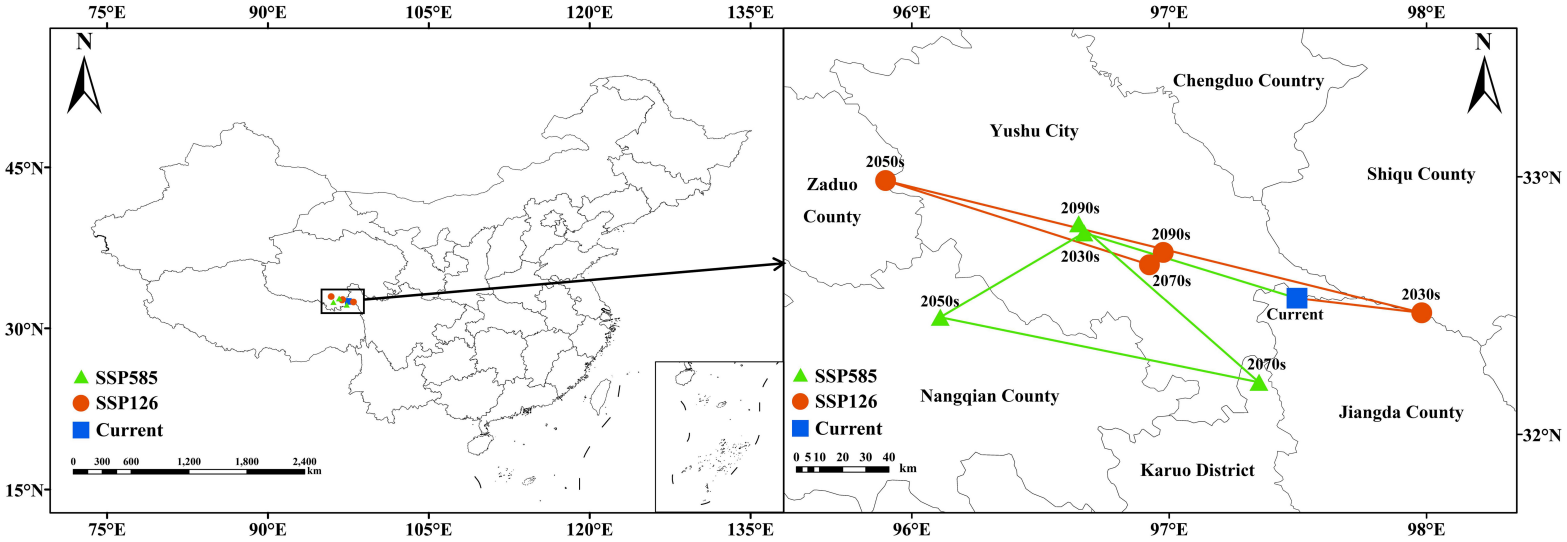
Migration trajectory of the geometric centroid of suitable areas for *A. pendulum* under future climate scenarios.

The MTSPS value was adopted as the critical threshold to distinguish between suitable and unsuitable areas for *A. pendulum*. ArcGIS Desktop software was employed to analyze the temporal migration characteristics of the centroid of the suitable habitat under the two emission scenarios (SSP126 and SSP585). Under current climatic conditions, the centroid of the suitable habitat for *A. pendulum* is situated in Jiangda County, Changdu City, Xizang Province. Starting from this location, the migration trajectory of the centroid has been plotted (**Figure 10**).

Under the SSP126 emission scenario, the centroid of the suitable habitat for *A. pendulum* will move 54.21 km southeastward to Shiqu County, Ganzi Tibetan Autonomous Prefecture, Sichuan Province during the 2021–2040 period. From 2041 to 2060, it will move 238.40 km northwestward to Zaduo County, Yushu Tibetan Autonomous Prefecture, Qinghai Province. During 2061-2080, the centroid will move another 119.58 km southeastward to Yushu City, which is also part of Yushu Tibetan Autonomous Prefecture in Qinghai Province. Finally, during 2081-2100, it will continue to move 7.99 km northeastward within the territory of Yushu City.

Under the SSP585 emission scenario, the centroid of the suitable habitat for *A. pendulum* will move 103.59 km northwestward to Yushu City in the 2021–2040 timeframe. In the 2041–2060 period, it will move 140.36 km southwestward to Nangqian County, Yushu Tibetan Autonomous Prefecture. During 2061-2080, the centroid will move an additional 71.96 km southeastward to Karuo District, Changdu City, and during 2081-2100, it will continue to move 96.22 km northwestward, thereby returning to Yushu City.

## 4 Discussion

### 4.1 The improvement of model optimization on prediction accuracy

This research centered on *A. pendulum*, a rare Chinese medicinal plant. We pruned redundant distribution points, eliminated multicollinearity among environmental variables, and systematically optimized the FC and RM of the MaxEnt model using the kuenm package. From 558 parameter combinations, the optimal solution (FC=LQ, RM = 1.2) was screened out, which reduced the model’s AICc value from 4618.5968 to 4403.5457 (ΔAICc=0). This optimization greatly decreased the model complexity while improving model goodness of fit, successfully resolving the problem associated with default parameters and providing a core guarantee for the scientific reliability of habitat prediction results.

### 4.2 Ecological driving effects of key environmental factors on the growth of *A. pendulum*

As a perennial herb with strong tolerance to cold and drought, *A. pendulum* naturally inhabits high-altitude regions across the Qinghai-Tibetan Plateau. Owing to its short growing season, September functions as a crucial period for nutrient accumulation in fleshy root tubers and the initiation of overwintering. Its phenological rhythms are predominantly regulated by seasonal thermal fluctuations, and its overall ecophysiological properties have evolved to adapt to the harsh alpine conditions, including low ambient temperatures, intensive solar radiation and substantial diurnal temperature swings (Li et al., 2024b). This species is physiologically vulnerable to high temperature, insufficient light and soil waterlogging. Geographically, its habitat range is an outcome of long-term adaptive evolution between intrinsic ecophysiological traits and extrinsic environmental drivers. Specifically, the ecological amplitude of major environmental parameters delineates its survival thresholds, and the synergism of these interacting factors constitutes the core mechanism underlying its present distribution.

Alt (contribution rate 35.5%) acts as the primary driving factor. Its suitable range of 1,930.9952-4,918.3328 m is highly consistent with the natural habitats of *A. pendulum*, which are mainly composed of alpine meadows, shrublands, and gravel slopes. The climatic conditions in high-altitude areas not only match the cold and drought-resistant physiological traits of *A. pendulum*, but also limit the colonization of competing species, creating a unique living environment for this species (Holdsworth et al., 2024). Srad_09 (contribution rate 29.2%) has a suitable range of 9853.2-15898.104 kJ·m^-2^·day^-1^, and directly affects the efficiency of nutrient accumulation in *A. pendulum*. As September represents the end of the growing season for this species, sufficient solar radiation promotes the translocation of photosynthetic products to the root system, storing energy for overwintering and germination in the next year. In contrast, insufficient radiation can lead to weak plant growth and reduced reproductive capacity (Suzuki et al., 2023). Bio_4 (contribution rate 17.7%), suitable range 463.7632-893.0871, affects plant survival by regulating phenological cycles. As a perennial herb, *A. pendulum* depends on seasonal temperature fluctuations to initiate rhythmic growth processes such as sprouting, flowering, and dormancy. Gentle temperature changes tend to cause phenological disorders at lower altitudes, while excessively drastic temperature fluctuations may induce frost damage in the extremely cold high-altitude regions (Chen et al., 2025b).

Furthermore, soil physicochemical properties (including bsat, bulk, and cec_clay) as well as prec_09 exert indirect impacts on the spatial distribution of *A. pendulum* by regulating the availability of soil moisture and nutrients. Soil water-retention capacity and aeration conditions govern the efficiency of root water absorption, whereas moderate rainfall in September mitigates drought stress during the late stage of the growing season (Pais et al., 2022). The combined synergistic effects of these edaphic and climatic factors collectively shape the distinctive habitat traits of *A. pendulum*, which are featured by high altitude, strong solar radiation, and stable temperature rhythms.

### 4.3 Distribution characteristics of suitable habitats for *A. pendulum* under current climate conditions

Under contemporary climatic scenarios, the overall suitable habitat extent for *A. pendulum* amounts to 2.08×10^6^ km^2^, representing 21.56% of China’s total territorial area. Its spatial distribution presents a pattern of concentrated cores in southwestern, northwestern and central China, accompanied by sporadic occurrence in peripheral regions. The high-suitable areas, covering 4.8×10^5^ km^2^, 5% of the total, is mainly concentrated in the transitional zone linking the eastern edge of the Qinghai-Tibet Plateau and the Sichuan Basin. Endowed with moderate elevation, stable climatic conditions and weak anthropogenic disturbance, this region acts as the core survival zone for wild *A. pendulum* populations (Paquette et al., 2006). The medium-suitable areas (3.8×10^5^ km^2^, 3.96%) expands outward from the core distribution area, whereas the low-suitable areas (1.22×10^6^ km^2^, 12.71%) spans the arid regions of northwestern China and the mountainous peripheries of southwestern China, which reflects the adaptive gradient of *A. pendulum* across diverse habitat types.

### 4.4 Patterns of habitat evolution for *A. pendulum* under future climate change

Under the two future emission scenarios, the overall suitable habitat area of *A. pendulum* displays a general decreasing tendency, yet the magnitude and structural characteristics of these alterations differ considerably. Under the SSP126 scenario, habitats of varying suitability levels exhibit distinct fluctuation patterns, with no unidirectional linear increase or decrease observed. Specifically, the unsuitable areas fluctuates only marginally; within the suitable habitat range, different suitability grades undergo mutual area shifts, and the total suitable habitat area initially rises, followed by a decline and a subsequent rebound. For the SSP585 scenario, the total suitable habitat area increases slightly solely during 2041-2060, and shows a continuous decreasing trend across all other periods, falling to its minimum value in the 2090s. The low-suitable areas contracts notably, while the high-suitable areas expands markedly, which reflects the habitat polarization induced by extreme climatic events under high-emission scenarios. The peripheral unsuitable areas shrink rapidly, while the core high-quality suitable zones expand modestly, as climatic conditions gradually become more congruent with the survival requirements of *A. pendulum*.

The migration dynamics of suitable habitats distinctly reflect the adaptive response of *A. pendulum* to climate change. Under the SSP126 scenario, the habitat centroid shifts back and forth among Changdu City, Ganzi Tibetan Autonomous Prefecture and Yushu City, with a total migration distance of 419.18 km, and always remains within the core distribution zone on the eastern margin of the Qinghai-Tibet Plateau. This pattern can be ascribed to the complex topographic conditions and strong climate-buffering capacity of this region (Sun et al., 2024b). In such a low-emission scenario, the temperature increase is relatively limited, and the core area maintains favorable hydrothermal conditions, resulting in only short-distance dispersal of peripheral populations (Zhang et al., 2019). Conversely, under the SSP585 scenario, the habitat centroid presents a remarkable migratory trend following a “northwest-southwest-southeast-northwest” route, with an overall migration distance of 412.13 km, and fluctuates mainly around Yushu City and Changdu City. This outcome indicates the frequent occurrence of extreme weather events under high-emission scenarios, the weakened climatic stability of core habitats, and the requirement for *A. pendulum* populations to undertake long-distance migration to avoid thermal stress (Balfagón et al., 2020).

### 4.5 Impacts of human factors on the future distribution of *A. pendulum*

Under the present study, model projections were established under an idealized framework that disregards anthropogenic disturbances and presupposes unimpeded population dispersal. Nevertheless, in practical natural settings, a series of factors including socioeconomic development, land-use transitions, governmental regulatory policies, and human-induced disturbances can substantially modify the realistic spatial distribution of suitable habitats for the species (Zhang et al., 2020). To begin with, anthropogenic activities spurred by economic growth directly invade and occupy the suitable habitats of *A. pendulum* located in low- to medium-elevation zones, which further triggers severe habitat fragmentation. Given that the eastern edge of the Qinghai-Tibet Plateau serves as the overlapping zone between the Sanjiangyuan Ecological Reserve and economic development sector, the risk of habitat degradation and loss in this region is particularly prominent. Secondly, shifts in land-use patterns, such as the transformation of alpine meadows into agricultural land and the construction of artificial forests, disturb soil physical structure and vegetation coverage, consequently disrupting the natural growth environment of *A. pendulum*. This impact is especially pronounced in low-suitable areas, where weaker ecological resilience renders habitats more susceptible to damage caused by land-use conversion.

Governmental policies and human interference exert dual effects on habitat stability. On one hand, the establishment of nature reserves effectively safeguards wild populations within high-suitable areas and preserves the integrity of core distribution areas. On the other hand, the high medicinal value of *A. pendulum* has triggered excessive exploitation of its wild resources. Even within suitable habitats, population abundance may drop sharply due to unregulated human harvesting. In addition, the absence of mature artificial cultivation technologies has further intensified pressure on wild resource stocks. At present, cultivated individuals cannot sufficiently substitute for wild *A. pendulum* resources, and market-driven collection activities will continue to threaten wild populations. In extreme cases, such disturbances may even result in habitat vacancy, where suitable areas no longer support viable wild populations.

## 5 Conclusion

In summary, this study applied the MaxEnt model optimized via the kuenm package to evaluate the potential distribution of *A. pendulum* in China under present and future climatic scenarios, and recognized alt, srad_09, and bio_4 as the dominant environmental determinants. Although the predictive accuracy has been elevated through multi−step model optimization, several limitations still exist. The model fails to consider biotic factors including interspecific competition, plant diseases and insect pests, and relies on the hypothesis of full population mobility, which is inconsistent with realistic ecological conditions. Moreover, the selected environmental variables do not cover indicators such as soil microbial communities and the frequency of extreme weather events, all of which restrict the completeness of the projection outcomes. Future research can be expanded in the following directions: first, verify the model predictions with field survey data and further optimize the environmental variable system; second, integrate anthropogenic disturbance indicators to build a dual−driver prediction model driven by “climate and human activities”; third, investigate the migration rate and adaptive capacity of *A. pendulum* populations, so as to assess its actual survival risks under the combined stress of climate change and human interference.

Based on the projected spatiotemporal dynamics of suitable habitats for *A. pendulum*, this study develops a differentiated conservation framework adapted to diverse habitat types and future climate scenarios. For core stable habitats, exclusive protected zones should be designated to limit large-scale wild harvesting and anthropogenic disturbances, and long-term ecological monitoring should be implemented to track population and habitat dynamics for the conservation of core wild germplasm resources. For expansion areas, baseline ecological surveys and buffer zone construction are urgently needed, with priority placed on protecting peripheral populations to preserve their unique genetic diversity and climate adaptability. For contraction areas, seasonal protection bans and stringent harvesting regulations are indispensable to prevent the overexploitation of wild populations. The promotion of standardized artificial cultivation can reduce dependence on wild resources, whereas habitat restoration and ecological corridor development can substantially improve habitat connectivity and gene flow among fragmented populations. Adaptive conservation measures are critical for coping with different climate change scenarios. Under low-emission scenarios, conservation efforts should focus on maintaining the integrity and connectivity of core habitats to guarantee stable population persistence. By comparison, high-emission scenarios necessitate a three-tier conservation strategy, including strict protection of core habitats, proactive conservation of expanded habitats, and targeted ecological intervention in degraded contraction habitats, to mitigate climate-induced habitat northward shifts and local habitat degradation. Furthermore, the integration of community co-management and ecological compensation mechanisms can reconcile ecological conservation with socioeconomic development, providing reliable scientific references for the long-term conservation and sustainable utilization of wild *A. pendulum* resources.

## Data Availability

The original contributions presented in the study are included in the article/**Supplementary Material**. Further inquiries can be directed to the corresponding authors.
